# Heterogeneous prognosis among *KIT* mutation types in adult acute myeloid leukemia patients with t(8;21)

**DOI:** 10.1038/s41408-018-0116-1

**Published:** 2018-08-07

**Authors:** Ya-Zhen Qin, Hong-Hu Zhu, Qian Jiang, Lan-Ping Xu, Hao Jiang, Yu Wang, Xiao-Su Zhao, Yan-Rong Liu, Xiao-Hui Zhang, Kai-Yan Liu, Xiao-Jun Huang

**Affiliations:** 1 Peking University People’s Hospital, Peking University Institute of Hematology, Beijing Key Laboratory of Hematopoietic Stem Cell Transplantation, Beijing, 100044 China; 2 Peking-Tsinghua Center for Life Sciences, Beijing, 100871 China

Acute myeloid leukemia (AML) with t(8;21) is a heterogeneous disease^1^. Therefore, additional prognostic factors are needed in order to make risk-adapted treatment approaches. *KIT* mutations are the most common mutations of t(8;21) AML patients, and a spectrum of mutations has been detected to date^2–6^. Limited by sample size or the screening method, previous studies have usually analyzed all types of mutations as a whole or just analyzed the most prevalent D816 mutation^2–7^. Thus, whether each type of mutation has similar adverse impacts remains unclear to date. A reflection is that the existence of the *KIT* mutation brings t(8;21) AML from low to intermediate risk regardless of mutation type in the National Comprehensive Cancer Network guidelines^8^, whereas European LeukemiaNet has provided no further recommendation for those with a *KIT* mutation^9^. Recently, Yui et al.^10^ showed that the D816 mutation had a poorer prognosis than other mutations. Thus, it is urgent to perform large-scale studies under modern treatment modes to comprehensively evaluate the prognoses of the individual *KIT* mutation types.

A total of 275 consecutive adult patients with t(8;21) AML who were diagnosed and received treatment in our institute from June 2005 to December 2017 were, retrospectively, evaluated. Totally, 150 patients (54.5%) were male. The median age of the patients at diagnosis was 36 (range: 16–69) years. As we previously reported, induction chemotherapy comprised 1–2 cycles of induction with the “3 + 7” regimen or the homoharringtonine, aclarubicin, and cytarabine regimen (homoharringtonine 2 mg/m^2^ per day, cytarabine 100 mg/m^2^, and aclarubicin 20 mg/day on days 1–7)^11,12^. Among the 263 patients achieving complete remission (CR), 142 received the intermediate-dose cytarabine-based chemotherapy, 13 received chemotherapy followed by autologous-hematopoietic stem cell transplantation (auto-HSCT), 108 received chemotherapy followed by allogeneic-HSCT (allo-HSCT, human leukocyte antigen-identical sibling donor, *n* = 43; matched unrelated donor, *n* = 7; haploidentical related donor, *n* = 58) as postremission therapy^13^. Dasatinib were used in some patients with *KIT* mutation if *RUNX1–RUNX1T1* reduction is less than 3-log after cycle 2 consolidation since 2015. Nine and one patients who relapsed after chemotherapy and auto-HSCT received allo-HSCT as salvage therapy. The study was approved by the Ethics Committee of the Peking University People’s Hospital. Informed consent was obtained from all subjects in accordance with the Declaration of Helsinki. The cutoff date for the follow-up was April 15, 2018. As we previously reported, the complementary DNA was used to amplify *KIT* exons 17 and 8 and sequencing^4^, and TaqMan-based real-time quantitative polymerase chain reaction technology was used to detect *RUNX1–RUNX1T1* transcript levels^11^. The survival functions were estimated using the Kaplan–Meier method and were compared using the log-rank test. The parameters with *P* < 0.20 by the univariate analysis were entered into a multivariate model using a Cox proportional hazards model to identify the most statistically significant parameters associated with relapse free survival (RFS) and overall survival (OS). The SPSS 16.0 software package (SPSS Inc., Chicago, IL) and GraphPad Prism 5 (GraphPad Software Inc., La Jolla, CA) were used for the data analysis.

The median follow-up time was 20 (2–93) months. The 3-year RFS and OS rates were 61.5% (95% confidence interval (CI), 53.9–68.2%) and 73.2% (95% CI, 67.3–80.4%), respectively. Overall, 114 patients (41.5%) had *KIT* mutations, and a total of 22 types of mutations were detected (Table 1). In all, 103 and 11 patients, respectively, had sole and compound mutations (combination of 2 types), and 104 (37.8%) and 14 (5.1%) patients had a *KIT* mutation in exon 17 and exon 8 (sole or compound), respectively. The most prevalent mutation was exon 17 D816 (57.0% of the patients with *KIT* mutations), followed by the exon 17 N822, exon 8 deletion–insertion and exon 17 D820 mutations (27.2, 12.3 and 4.4%). The one-course and two-course CR rates were similar between the patients with *KIT* mutations and no mutation (*P* = 1.0 and 0.45). Patients with a *KIT* mutation had significantly lower 3-year RFS and OS rates than those with no mutation (RFS: *P* = 0.0002, 49.3% [95% CI: 37.0–60.5%] vs. 69.7% [95% CI 59.9–77.6%]; OS: *P* = 0.0055, 67.1% [95% CI: 55.0–76.6%] vs. 77.6% [95% CI: 68.3–84.5%]). Patients with sole D816V, D816Y, and D816H mutation had similar 3-year RFS and OS rates (*P* = 0.57 and 0.087). Patients with a sole D816 mutation had significantly lower 3-year RFS and OS rates than those with no mutation (RFS, *P* < 0.0001, 33.7% [95% CI: 17.3–50.9%] vs. 69.7% [95% CI: 59.9–77.6%], Fig. 1a; OS, *P* < 0.0001, 54.9% [95% CI: 37.9–69.1%] vs. 77.6% [95% CI: 68.3–84.5%], Fig. 1b); Similar results existed if the patients who underwent allo-HSCT were censored at the time of transplantation (RFS: *P* < 0.0001, 19.4% [95% CI: 1.6–52.3%] vs. 57.7% [95% CI: 43.8–69.3%], Fig. 1c; OS: *P* = 0.0003, 53.7% [95% CI: 23.9–76.3%] vs. 77.0% [95% CI: 63.2–86.2%], Fig. 1d). In addition, the 3-year RFS and OS rates were similar among the patients with the sole N822 mutation, the exon 8 mutation and no mutation (RFS: *P* = 0.47, 69.6% [95% CI: 46.1–84.4%] vs. 88.9% [95% CI: 43.3–98.4%] vs. 69.7% [95% CI: 59.9–77.6%], Fig. 1a; OS: *P* = 0.70, 71.9% [95% CI: 42.7–88.0%] vs. 83.3% [95% CI: 27.3–97.4%] vs. 77.6% [95% CI: 68.3–84.5%], Fig. 1b). Likewise, the 3-year RFS and OS rates were similar if censoring at the time of transplantation (RFS: *P* = 0.36, 52.6% [95% CI: 18.5–78.3%] vs. 80.0% [95% CI: 20.4–96.9%] vs. 57.7% [95% CI: 43.8–69.3%], Fig. 1c; OS: *P* = 0.32, 0 [95% CI: 0–0] vs. 75.0% [95% CI: 12.8–96.1%] vs. 77.0% [95% CI: 63.2–86.2%], Fig. 1d). Because of the similar prognoses for the N822 and exon 8 mutations compared to no mutation, five patients with sole or compound D820 mutation (Table 1) were analyzed together. Patients with the D820 mutation had significantly lower 3-year RFS rates than those with no mutation despite of no censoring or censoring (no censoring: *P* = 0.0050, 20.0% [95% CI: 0.8–58.2%] vs. 69.7% [95% CI: 59.9–77.6%], Fig. 1a; censoring: *P* < 0.0001, 0% [95% CI: 0–0%] vs. 57.7% [95% CI: 43.8–69.3%], Fig. 1c). However, the D820 mutation had no impact on OS (*P* = 0.73 and 0.72, Fig. 1b and d).

**Table 1:** KIT mutation patterns

Type of mutation	Number of patients (%)
*Sole mutation*	103 (90.4%)
Exon 17	92 (80.7%)
R815_D816delinsK	1 (0.9%)
R815_D816insT	1 (0.9%)
R815_D816insIR	1 (0.9%)
D816A	1 (0.9%)
D816H	8 (7.0%)
D816V	37 (32.5%)
D816Y	10 (8.8%)
D820G	3 (2.6%)
N822K	28 (24.6%)
N822Y	1 (0.9%)
A829P	1 (0.9%)
Exon 8	11 (9.6%)
T417_D419DelinsI	1 (0.9%)
T417_D419delinsY	1 (0.9%)
T417_R420DelinsG	1 (0.9%)
Y418delinsFFW	1 (0.9%)
Y418_D419delinsP	1 (0.9%)
Y418_R420delinsSW	1 (0.9%)
Y418_L421delinsTRVY	1 (0.9%)
D419del	1 (0.9%)
D419_R420delinsK	2 (1.8%)
D419_L421DelinsVEV	1 (0.9%)
*Compound mutations*	11 (9.6%)
D816V + D816H	2 (1.8%)
D816V + D816Y	3 (2.6%)
D816V + D820G	1 (0.9%)
D816V + D419del	1 (0.9%)
D816V + T417_L421delinsLPRF	1 (0.9%)
D816Y + N822K	1 (0.9%)
D820G + N822K	1 (0.9%)
D820G + D419del	1 (0.9%)
*Total*	114 (100%)

**Fig. 1:**
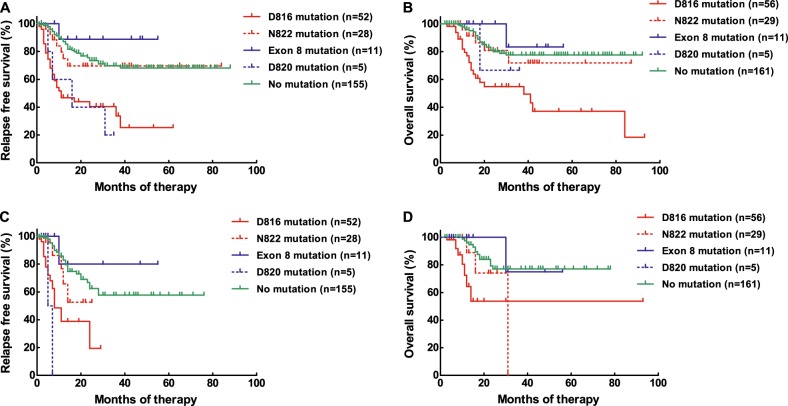
RFS and OS of patients grouped by *KIT* mutation status and type. **a** RFS, no censoring. **b** OS, no censoring. **c** RFS, censoring at the time of allo-HSCT. **d** OS, censoring at the time of allo-HSCT

Next, patients with *KIT* D816 and D820 mutations were defined as the D816/D820 mutation group (*n* = 70, 25.5%), whereas the N822 and exon 8 mutation and no mutation were defined as the N822/exon 8/no mutation group (*n* = 201, 73.1%). Patients with the D816/D820 mutation had significantly lower 3-year RFS and OS rates than those with the N822/exon 8/no mutation (RFS: *P* < 0.0001, 33.1% [95% CI: 18.7–48.2%] vs. 70.5% [95% CI: 61.9–77.5%]; OS: *P* < 0.0001, 60.5% [95% CI: 45.3–72.7%] vs. 77.2% [95% CI: 68.8–83.6%]). Similar results existed when censoring (RFS: *P* < 0.0001, 0% [95% CI: 0–0%] vs. 57.9% [95% CI: 45.2–68.7%]; OS: *P* = 0.0002, 58.5% [95% CI: 31.9–77.7%] vs. 73.6% [95% CI: 60.3–83.1%]). Multivariate analyses showed that the *KIT* D816/D820 mutation, a <3-log reduction in the *RUNX1–RUNX1T1* transcript levels at cycle 2 consolidation and treatment with chemotherapy only/auto-HSCT were independent adverse prognostic factors for both RFS and OS (Table S1).

In accordance with the majority of previous studies^2–7^, we confirmed that both the *KIT* mutation and the *KIT* D816 mutation were significantly associated with lower RFS and OS rates in adult t(8;21) AML. We also showed that the three common D816 mutations had similar clinical impacts. Furthermore, we demonstrated that the N822 and exon 8 mutations had similar RFS and OS rates compared to no mutation, whereas the D820 mutation had a significantly higher relapse probability than no mutation. The results implied that we should stratify the patients not only according to the existence of the *KIT* mutation but also according to the type of mutation. After regrouping, the *KIT* D816/D820 mutation was shown to be an independent adverse prognostic factor for both RFS and OS. The multivariate analyses result reflected that the pretreatment factor, treatment response and treatment modality were all relevant to the outcome of t(8;21) AML.

Consistent with the current clinical results, animal and in vitro studies show a functional difference between *KIT* mutations. Nick et al.^14^ used a murine model to illustrate that *KIT*^D814V^ promoted a more varied and aggressive leukemic phenotype than *KIT*^T417IΔ418–419^ when coexpressed with *RUNX1–RUNX1T1*. Omori et al. demonstrated that in addition to the common JAK/STAT signaling pathway, the D816V mutation activated SRC family kinases, whereas N822K activated the MAPK pathway. The consequence was that D816V had a greater cell-proliferative and antiapoptotic ability than the N822K mutation^15^.

The limitation was that this was a retrospective study. The treatment regimens were not uniform. Furthermore, we could not analyze the synergistic impact of the individual *KIT* mutations with other gene mutations due to lack of data.

In conclusion, the individual *KIT* mutations had distinct prognoses in adult t(8;21) AML. Exon 17 D816 and D820 mutation had an adverse prognosis, whereas the exon 17 N822 and exon 8 mutation had a similar prognosis to no mutation. This result is helpful for a more precise stratification and for directing the appropriate treatment in t(8;21) AML. Multicenter prospective studies with a large sample size are warranted.

## Electronic supplementary material

Table S1

## References

[1] Marcucci G (2005). Prognostic factors and outcome of core-binding factor acute myeloid leukemia patients with t(8;21) differ from those of patients with inv(16): a Cancer and Leukemia Group B study. J. Clin. Oncol..

[2] Paschka P (2006). Adverse prognostic significance of KIT mutations in adult acute myeloid leukemia with inv(16) and t(8;21): a Cancer and Leukemia Group B Study. J. Clin. Oncol..

[3] Duployez N (2016). Comprehensive mutational profiling of core-binding factor acute myeloid leukemia. Blood.

[4] Qin YZ (2014). Prevalence and prognostic significance of c-KIT mutations in core-binding factor acute myeloid leukemia: a comprehensive large-scale study from a single Chinese center. Leuk. Res..

[5] Cher CY (2016). Next-generation sequencing with a myeloid gene panel in core-binding factor AML showed KIT activation loop and TET2 mutations predictive of outcome. Blood Cancer J..

[6] Jang W (2016). Significance of KIT exon 17 mutation depends on mutant level rather than positivity in core-binding factor acute myeloid leukemia. Blood Cancer J..

[7] Schnittger S (2006). KIT D816 mutations in AML1-ETO-positive AML are associated with impaired event-free and overall survival. Blood.

[8] National Comprehensive Cancer Network clinical practice guidelines in oncology (NCCN Guidelines): acute myeloid leukemia. Version 1. 2018. 10.6004/jnccn.2018.0071 30181422

[9] Döhner H (2017). Diagnosis and management of AML in adults: 2017 ELN recommendations from an international expert panel. Blood.

[10] Yui S (2017). D816 mutation of the KIT gene in core-binding factor acute myeloid leukemia is associated with poorer prognosis than other KIT gene mutations. Ann. Hematol..

[11] Zhu HH (2013). MRD-directed risk stratification treatment may improve outcomes of t(8;21) AML in the first complete remission: results from the AML05 multicenter trial. Blood.

[12] Zhu HH (2016). Homoharringtonine, aclarubicin and cytarabine (HAA) regimen as the first course of induction therapy is highly effective for acute myeloid leukemia with t (8;21). Leuk. Res..

[13] Qin YZ (2016). Low WT1 transcript levels at diagnosis predicted poor outcomes of acute myeloid leukemia patients with t(8;21) who received chemotherapy or allogeneic hematopoietic stem cell transplantation. Chin. J. Cancer.

[14] Nick HJ (2012). Distinct classes of c-Kit-activating mutations differ in their ability to promote RUNX1-ETO-associated acute myeloid leukemia. Blood.

[15] Omori I (2017). D816V mutation in the KIT gene activation loop has greater cell-proliferative and anti-apoptotic ability than N822K mutation in core-binding factor acute myeloid leukemia. Exp. Hematol..

