# Supplementary material for: Heterogeneous prognosis among KIT mutation types in adult acute myeloid leukemia patients with t(8;21)
Source: Blood Cancer J. 2018 Aug 7;8(8):76. doi: 10.1038/s41408-018-0116-1 (PMC6081455; doi:10.1038/s41408-018-0116-1)
Supplement: Supplementary file 1 — Table S1 [file 41408_2018_116_MOESM1_ESM.docx]

**Table S1**. Multivariate analyses of RFS and OS

| Variable | RFS | |  | OS | |
| --- | --- | --- | --- | --- | --- |
|  | HR (95% CI) | *P* value |  | HR (95% CI) | *P* value |
| *KIT* mutation ( D816/D820 vs N822/exon 8/no) | 6.2 (3.5–10.9) | <0.001 |  | 3.5 (1.8–6.8) | <0.001 |
| Log reduction of *RUNX1-RUNX1T1* transcript levels after cycle 2 consolidation (< 3 vs ≥ 3) | 4.3 (2.5–7.3) | <0.001 |  | 2.4 (1.2–4.6) | 0.010 |
| Treatment modality ( chemotherapy only/auto-HSCT vs allo-HSCT) | 7.6 (4.0–14.2) | <0.001 |  | 2.4 (1.2–4.8) | 0.012 |
